# PSMA PET in Lymph Node Staging of Prostate Cancer: From Diagnostic Accuracy to Clinical Decision-Making

**DOI:** 10.3390/cancers18132094

**Published:** 2026-06-27

**Authors:** Francesco Esperto, Arianna Pischetola, Matteo Bauckneht, Jacopo Passoni, Antonio Testa, Stefania Ferretti, Stefano Puliatti, Sebastiano Buti, Roberto Mario Scarpa, Davide Campobasso, Rocco Papalia

**Affiliations:** 1 Department of Urology, Fondazione Policlinico Universitario Campus Bio-Medico of Rome, 00128 Rome, Italy; francescoesperto@gmail.com (F.E.); robertomarioscarpa1@gmail.com (R.M.S.); rocco.papalia@policlinicocampus.it (R.P.); 2 Research Unit of Urology, Department of Medicine and Surgery, Università Campus Bio-Medico di Roma, Via Alvaro del Portillo 21, 00128 Roma, Italy; 3 Nuclear Medicine, Department of Health Sciences (DISSAL), University of Genova, 16132 Genova, Italy; matteo.bauckneht@gmail.com (M.B.); jacopopassoni02@gmail.com (J.P.); 4 IRCCS Azienda Ospedaliera Metropolitana, 16132 Genova, Italy; 5 Department of Urology, San Camillo De Lellis Hospital, ASL of Rieti, 02100 Rome, Italy; antonio.testa@asl.rieti.it (A.T.); d.campobasso@virgilio.it (D.C.); 6 Urology Unit, Azienda Ospedaliero-Universitaria of Modena, University of Modena and Reggio Emilia, 41125 Modena, Italy; stefaniaferretti@icloud.com (S.F.); stefano.puliatti@unimore.it (S.P.); 7 Medical Oncology Unit, University Hospital of Parma, 43126 Parma, Italy; sebastiano.buti@unipr.it; 8 Ph.D. Program in Learning Sciences and Digital Technologies, University of Modena and Reggio Emilia, 41125 Modena, Italy

**Keywords:** prostate cancer, PSMA PET, lymph node staging

## Abstract

Lymph node involvement in prostate cancer has major prognostic and therapeutic implications, yet conventional imaging based on size and morphology remains limited in detecting small-volume metastatic disease. Although extended pelvic lymph node dissection is considered the reference standard for nodal staging, it is invasive, associated with morbidity, and primarily diagnostic in intent. Prostate-specific membrane antigen positron emission tomography (PSMA PET) has reshaped staging by enabling molecular detection of nodal metastases, consistently demonstrating superior accuracy compared with conventional imaging. This narrative review critically evaluates the role of PSMA PET in lymph node staging, with a primary focus on primary diagnosis and selected considerations on biochemical recurrence, focusing not only on diagnostic accuracy but on clinical utility and decision-making. PSMA PET shows high specificity but moderate sensitivity for pelvic nodal metastases, with reduced performance for micrometastatic disease; therefore, a negative scan cannot reliably exclude nodal involvement in high-risk patients. Evidence indicates frequent stage migration and management changes, including refinement of surgical planning, radiotherapy target delineation, and treatment intensification strategies. However, most pivotal therapeutic trials were based on conventional imaging, and long-term outcome data validating PSMA PET-guided treatment adaptations remain limited. We discuss biological rationale, radiotracer characteristics, interpretation frameworks, guideline perspectives, real-world variability in adoption, and current limitations, including false-positive findings, PSMA heterogeneity, and lack of universal standardization. Rather than replacing established staging paradigms, PSMA PET should be integrated within a comprehensive, risk-adapted framework. Ongoing prospective trials will clarify whether molecularly defined nodal staging translates into improved oncologic outcomes and will determine its definitive role in contemporary prostate cancer management.

## Simple Summary

Lymph nodes are important in prostate cancer since their involvement can radically change prognosis and treatment. Standard scans, such as CT and MRI, often rely on lymph node size and may miss small deposits of cancer. Prostate-specific membrane antigen positron emission tomography (PSMA PET) is an imaging technique that can detect prostate cancer based on molecular features rather than size alone. This review elaborates how this scan can help physicians assess lymph node disease, guide surgery or radiotherapy planning, and support more personalized treatment decisions. It also highlights important limitations, including false-positive findings, missed microscopic disease, differences between imaging tracers, and the need to interpret results together with clinical risk. Overall, this review aims to help clinicians and researchers use this imaging tool more effectively while emphasizing that it should complement, not replace, established staging methods.

## 1. Introduction

### Lymph Node Staging in Prostate Cancer: Current Evidence and Guideline Perspectives

Accurate lymph node staging plays a central role in prostate cancer management due to nodal involvement having major implications for both prognosis and treatment selection. Pelvic lymph node metastases (N1) are associated with higher risks of biochemical recurrence and cancer-specific mortality, whereas extra-pelvic nodal disease (M1a) usually indicates systemic spread and the need for more intensive therapy [1,2,3,4]. Conventional imaging with CT and MRI relies on nodal size and morphology, which limits sensitivity for small-volume metastases and can result in both under- and over-staging [5]. In current practice, the indication for extended pelvic lymph node dissection is guided by validated nomograms (e.g., Briganti, MSKCC, Gandaglia) that estimate the individual probability of nodal involvement, which support risk-adapted selection for lymphadenectomy but remain limited by their reliance on clinicopathologic variables rather than direct detection of metastatic disease [6,7]. As a result, extended pelvic lymph node dissection (ePLND) has historically been regarded as the reference standard for nodal staging in patients undergoing radical prostatectomy, despite its invasiveness and potential morbidity. However, while its role remains primarily diagnostic, a clear therapeutic survival benefit has not been consistently demonstrated. Moreover, the diagnostic yield of ePLND varies with the extent of dissection and pathological assessment, highlighting its limitations as a universal staging approach [8,9].

Prostate-specific membrane antigen positron emission tomography (PSMA PET) has rapidly changed prostate cancer imaging by enabling molecular staging based on target expression rather than lymph node size alone. Across multiple prospective and retrospective studies, PSMA PET has consistently shown superior sensitivity and specificity compared with conventional imaging for detecting both pelvic nodal and distant metastases [10]. This advantage was clearly demonstrated in the randomized proPSMA trial, in which men with high-risk prostate cancer underwent either PSMA PET/CT or conventional imaging; PSMA PET provided significantly greater staging accuracy and led to more frequent changes in clinical management [11]. Follow-up analyses of the proPSMA trial have further demonstrated the prognostic value of PSMA PET-based nodal staging. PSMA PET-defined nodal status was significantly associated with treatment failure, whereas nodal staging based on conventional imaging did not show prognostic significance, highlighting the ability of PSMA PET to better stratify patients according to oncologic risk [12]. These prognostic findings are further corroborated by a post hoc analysis of the surgical cohort from the phase III trial by Hope et al., in which presurgical [^68^Ga]Ga-PSMA-11 PET/CT-defined nodal status, particularly the presence of PSMA-nonlocalized disease, was a significant independent predictor of biochemical recurrence-free survival [13]. More broadly, interim analyses from the international PROMISE-PET Registry, which includes over 11,000 patients across multiple centers, have demonstrated that PSMA PET-derived staging parameters, captured through the standardized miTNM classification are independently associated with overall survival across the entire disease spectrum, from primary staging through biochemical recurrence to metastatic castration-resistant disease [14,15]. These findings support the concept that PSMA PET provides not only improved diagnostic accuracy but also clinically meaningful prognostic stratification.

Major professional societies have increasingly incorporated PSMA PET into clinical guidelines, but differ in the strength and clinical implications of their recommendations. The EAU guidelines give a strong recommendation for PSMA PET/CT for initial staging in patients with high-risk localized or locally advanced prostate cancer, reflecting its superior accuracy for nodal and distant staging compared with conventional imaging [2,3]. The National Comprehensive Cancer Network (NCCN), instead, states that PSMA PET/CT or PSMA-PET/MRI, when available, can be considered as an alternative to CT, MRI, and bone scans for initial staging in unfavorable intermediate-, high-, and very high-risk disease, recommendations are category 2A unless otherwise specified [16]. The European Society for Medical Oncology (ESMO) likewise acknowledges the higher sensitivity and specificity of PSMA PET compared with conventional imaging but emphasizes that this has not been shown to improve clinical outcomes and considers the current evidence insufficient to issue a formal recommendation for its routine use in initial staging. ESMO further cautions that patients with localized disease on standard imaging should not be denied radical local treatment solely because metastatic lesions are identified on novel imaging techniques [17]. Despite broad agreement regarding its diagnostic advantages, guidelines continue to differ with respect to patient selection, timing of imaging, and how PSMA PET findings should be incorporated into treatment pathways. Real-world data suggest that the application of PSMA PET does not fully mirror guideline recommendations, with variability in patient selection, timing of imaging, and its role within staging pathways across institutions [18]. This variability highlights the persistent gap between the high diagnostic accuracy of PSMA PET and the currently limited outcome-based evidence supporting PET-driven treatment decisions.

Another interesting issue is the different perception about the use and utility of PSMA PET between clinicians and nuclear medicine physicians, as reported by a recent Italian survey. In this paper the Authors highlighted that only 50% and 20% of clinicians use PSMA PET for primary staging in high risk and intermediate risk patients, respectively. This difference may also be related to an uneven availability of PSMA PET with subsequent longer waiting times to perform a PET than conventional imaging modalities. This is in sharp contrast with 87% of nuclear medicine physicians considering PSMA PET as the preferred staging test [19]. These findings are further supported by additional national survey data demonstrating substantial heterogeneity in the adoption of PSMA PET across institutions, influenced by factors such as radiotracer availability, access to nuclear medicine infrastructure, and variability in its integration into clinical decision-making pathways [18].

Our aim is not simply to summarize diagnostic performance metrics, but to critically examine the clinical utility of PSMA PET for lymph node staging in prostate cancer. We focus on when PSMA PET should be used, how its findings should be interpreted across different clinical scenarios, and the extent to which it meaningfully influences treatment decisions. We also address current limitations, unresolved controversies, and emerging directions, with the goal of offering a balanced and clinically relevant framework for integrating PSMA PET into contemporary prostate cancer care. Given the nature of the narrative review, the available evidence is interpreted according to its clinical purpose and evidentiary strength, with prospective trials and histopathologically validated studies being prioritized and retrospective data are used mainly to contextualize the data.

## 2. Materials and Methods

### 2.1. Biological Rationale of PSMA and Molecular Basis of Imaging

Prostate-specific membrane antigen (PSMA) is a type II transmembrane glycoprotein that is markedly overexpressed in prostate cancer cells compared with most normal tissues. Its high, and prostate-restricted, expression among solid tumors has made PSMA one of the most extensively validated biomarkers for diagnostic imaging in prostate cancer. PSMA expression correlates with tumor grade and burden, with higher levels often observed in more aggressive disease. Although expression is heterogeneous, the overall prevalence and intensity of PSMA expression in prostate cancer underpin its value as a molecular imaging target [20,21,22].

At the molecular level, PSMA exhibits folate hydrolase (glutamate carboxypeptidase II) activity and is expressed on the external surface of tumor cells, allowing it to be targeted by small-molecule inhibitors labeled with positron-emitting isotopes such as gallium-68 or fluorine-18. These PSMA-targeted radioligands bind with high affinity to the extracellular domain of PSMA and undergo receptor-mediated internalization, resulting in intracellular tracer accumulation and high tumor-to-background contrast on PET imaging [20,23,24].

This imaging approach is fundamentally different from conventional modalities such as CT or MRI, which rely primarily on anatomical features like lymph node size and morphology. By targeting a tumor-associated antigen at the molecular level, PSMA PET provides superior sensitivity and specificity compared with conventional imaging for detecting prostate cancer metastases, including small lymph node deposits that may be missed on standard imaging [25].

### 2.2. PSMA Radiotracers and Their Implications for Lymph Node Staging

The PSMA radiotracers most used in clinical practice for prostate cancer staging include [^68^Ga]Ga-PSMA-11 and the ^18^F-labeled compounds, primarily [^18^F]PSMA-1007, [^18^F]rhPSMA-7.3, [^18^F]DCFPyL, and [^18^F]JK-PSMA-7. Although all target the same prostate-specific membrane antigen, they differ in physical properties and biodistribution, which can affect the detection of pelvic and retroperitoneal lymph node metastases. The main characteristics of the most used PSMA radiotracers and their implications for lymph node staging are summarized in Table 1. Overall, comparative studies suggest broadly similar diagnostic performance between ^18^F- and ^68^Ga-based tracers for nodal staging, with some evidence of slightly higher lesion detection rates or SUV values with [^18^F]-labeled agents, although without consistent or definitive superiority across clinical settings [26,27].

**Figure 1:**
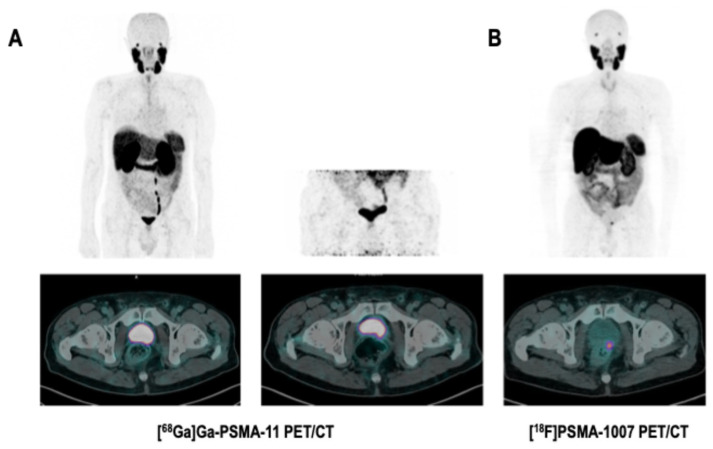
Intra-individual comparison of PSMA PET/CT using different radiotracers in a patient with biochemical recurrence after radical prostatectomy. Clinical history: robot-assisted radical prostatectomy with extended pelvic lymph node dissection (RALP + ePLND) in 2019; biochemical recurrence in 2020. Panel (**A**): [^68^Ga]Ga-PSMA-11 PET/CT performed at a PSA level of 0.8 ng/mL. Standard whole-body imaging with additional delayed pelvic acquisition after forced diuresis did not demonstrate definite evidence of local recurrence, with urinary tracer activity partially limiting evaluation of the prostate bed [32]. Panel (**B**): [^18^F]PSMA-1007 PET/CT performed two months later at a PSA level of 0.95 ng/mL. Due to minimal urinary excretion, a focal uptake consistent with local recurrence in the prostate bed is clearly detectable, which was not identified on the prior [^68^Ga]Ga-PSMA-11 scan.

[^68^Ga]Ga-PSMA-11 is the most extensively validated PSMA tracer and remains widely used, particularly in centers with access to on-site local generators. It has a relatively short physical half-life of approximately 68 min and is produced using a germanium/gallium generator, enabling local synthesis but limiting centralized distribution. Its diagnostic performance is driven by high binding affinity to PSMA, which is overexpressed in prostate cancer cells, with subsequent rapid internalization and intracellular retention of the radiotracer. These pharmacokinetic properties, combined with relatively fast clearance from non-target tissues, allow for high lesion-to-background contrast and detection of small-volume disease [28]. In the context of lymph node staging, the ability of [^68^Ga]Ga-PSMA-11 for detecting pelvic and retroperitoneal lymph node metastases is well established, with consistently high specificity (95% for nodal involvement) and high positive predictive value for BCR (92%), although sensitivity (40%) declines for sub-centimeter nodal disease [29,30,31]. A key limitation is its predominant renal excretion, resulting in significant tracer accumulation within the urinary tract and bladder. This may obscure adjacent pelvic structures, particularly lymph nodes located near the bladder or prostatic bed, thereby potentially reducing diagnostic sensitivity in this anatomically critical region [28] (see an emblematic example in Figure 1).

^18^F-labeled PSMA radiotracers, including [^18^F]PSMA-1007 and [^18^F]DCFPyL, offer several practical and imaging advantages compared with ^68^Ga-labeled tracers. These tracers have a longer physical half-life of approximately 110–120 min, allowing centralized cyclotron-based production. Their overall diagnostic performance is broadly comparable to that of [^68^Ga]Ga-PSMA-11, with similarly high specificity (96 to 99%) for nodal metastases and detection rates (85 to 86% for BCR) across both primary staging and biochemical recurrence settings. From a pharmacokinetic perspective, 18F-labeled tracers demonstrate distinct biodistribution patterns. [^18^F]PSMA-1007 is characterized by predominantly hepatobiliary clearance with minimal urinary excretion, whereas [^18^F]DCFPyL exhibits partial renal excretion but with rapid clearance from the urinary tract, which can facilitate clearer assessment of pelvic lymph nodes near the bladder and ureters, especially in the obturator, internal iliac, and presacral regions. This may improve detection in anatomically challenging areas compared with ^68^Ga-labeled tracers. However, important differences exist between these agents: [^18^F]PSMA-1007 has been associated with a higher incidence of nonspecific uptake (sensitivity is approximately 42 to 61%), particularly in bone and soft tissues, which may complicate interpretation and necessitate careful correlation with anatomical imaging, whereas [^18^F]DCFPyL demonstrates a biodistribution more comparable to [^68^Ga]Ga-PSMA-11, with generally fewer nonspecific findings reported in some series [26,28,33,34].

More recently, the radiohybrid PSMA ligand [^18^F]rhPSMA-7.3 has received FDA approval for PET imaging of PSMA-positive lesions in men with prostate cancer, based on the phase 3 LIGHTHOUSE and SPOTLIGHT trials [35,36], and has been included in the NCCN guidelines [37]. [^18^F]rhPSMA-7.3 demonstrates high PSMA binding affinity, favorable biodistribution with reduced urinary excretion compared with [^68^Ga]Ga-PSMA-11, and a longer physical half-life, allowing centralized cyclotron-based production. In the LIGHTHOUSE trial, which enrolled 335 newly diagnosed patients with unfavorable intermediate- to very high-risk disease, [^18^F]rhPSMA-7.3 PET/CT achieved high specificity (93–97%) for pelvic lymph node detection validated against histopathology, although sensitivity ranged between 23% and 30% [35]. In the SPOTLIGHT trial, the agent demonstrated high detection rates (88%) in patients with biochemical recurrence, with a verified pelvic lymph node detection rate of 28% in post-prostatectomy patients [36]. EMA approval is currently pending. The relatively low sensitivity observed in LIGHTHOUSE should be interpreted in line with the broader limitations of PSMA PET for pelvic nodal staging, particularly the histopathology-based detection of small-volume or micrometastatic nodal disease below PET resolution.

Another [^18^F]-labeled PSMA ligand gaining clinical interest is [^18^F]JK-PSMA-7 (2-methoxy [^18^F]-DCFPyL), which has demonstrated high target-to-background ratios in preclinical comparisons and reported broadly comparable clinical utility to [^68^Ga]Ga-PSMA-11 in intra-individual comparison studies. In patients with biochemical recurrence, [^18^F]JK-PSMA-7 PET/CT achieved overall detection rates of approximately 85%, with PSA-stratified detection rates ranging from 54.5% (PSA < 0.5 ng/mL) to 90.9% (PSA > 2 ng/mL). Its combined renal and hepatobiliary excretion may offer a favorable pelvic assessment. Although not yet approved by major regulatory agencies through a centralized marketing authorization, [^18^F]JK-PSMA-7 has recently been included in the European Pharmacopoeia (monograph 3205, Issue 12.2, implementation date April 2026), which provides a standardized framework for its production and quality control at hospital radiopharmacies across European institutions. This regulatory milestone is expected to facilitate broader clinical adoption.

In current clinical practice, no single PSMA radiotracer has shown unequivocal superiority for lymph node staging. Tracer selection is therefore largely guided by the clinical context, disease distribution, and institutional experience. [^68^Ga]-labeled tracers can be produced without cyclotron infrastructure. [^18^F]-labeled agents require cyclotron-based production, which can be performed either on-site at centers equipped with a cyclotron or at centralized facilities with subsequent distribution to satellite centers, an approach facilitated by the longer physical half-life of [^18^F] (~110 min vs. ~68 min for [^68^Ga]) [26,27]. Awareness of tracer-specific characteristics, particularly urinary excretion patterns, and the potential for benign uptake, is essential for accurate interpretation of pelvic and retroperitoneal lymph node findings and to minimize false-positive results that could lead to unnecessary interventions.

### 2.3. Diagnostic Performance of PSMA PET in Primary Lymph Node Staging

PSMA PET has significantly improved the non-invasive assessment of lymph node metastases in newly diagnosed prostate cancer, particularly in patients with intermediate- and high-risk disease. Multiple systematic reviews and prospective studies comparing PSMA PET/CT with histopathology from radical prostatectomy and extended lymph node dissection consistently report high specificity but only moderate sensitivity for pelvic nodal staging [38,39]. In the largest prospective cohort to date, [^68^Ga]Ga-PSMA-11 PET/CT achieved a specificity of approximately 95% and a sensitivity of around 40% for detecting pelvic lymph node metastases when benchmarked against histopathology, underscoring its high positive predictive value but limited ability to reliably exclude microscopic disease [31]. This limitation is particularly relevant in high-risk surgical candidates, in whom the probability of nodal involvement remains substantial.

Meta-analytic data indicate that PSMA PET is more sensitive for larger lymph node metastases (≥5 mm) and outperforms conventional cross-sectional imaging with CT or MRI. Pooled estimates typically show sensitivity in the range of ~50–80% and specificity of ≥90%, although substantial heterogeneity across studies remains [40,41]. Single-institution series and meta-analyses also report a high negative predictive value for nodal metastases in selected intermediate-risk patients, suggesting a potential role for PSMA PET in guiding decisions about the necessity and extent of lymph node dissection [38,42,43].

Despite these promising results, sensitivity remains suboptimal for micrometastatic disease, representing a key limitation for preoperative staging. Small-volume nodal metastases frequently fall below the spatial resolution of PET imaging, even with PSMA-targeted tracers [44]. This limitation is further supported by data from the prospective SALT trial, in which median tumor size of false-negative pelvic lymph node metastases on [^18^F]DCFPyL PET/CT was only 1.5 mm, compared with 5.5 mm for correctly identified metastases, underscoring the inherent spatial resolution constraints of current PET technology [45]. Consequently, current evidence supports the use of PSMA PET as a strategic adjunct to clinical and nomogram-based risk assessment rather than as a standalone replacement for histopathologic evaluation, particularly when surgical lymph node dissection is being considered.

### 2.4. Clinical Impact of PSMA PET on Lymph Node Management

**Figure 2:**
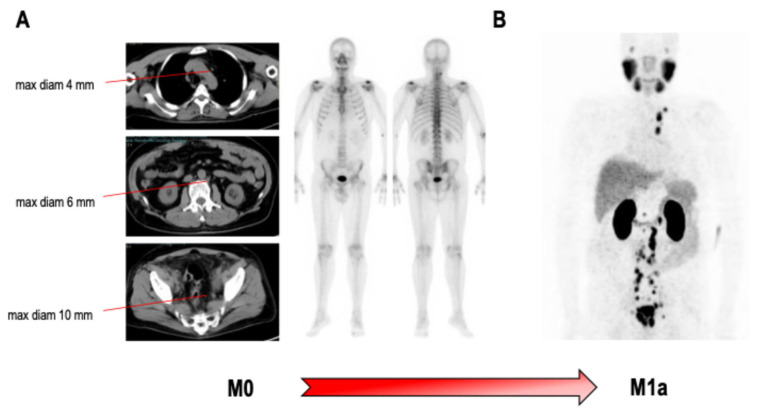
Stage migration with PSMA PET/CT in a 65-year-old man with high-risk prostate cancer (cT3, Gleason score 4 + 4, initial PSA 42 ng/mL). (**A**) Conventional imaging: contrast-enhanced CT (left) shows three lymph nodes of borderline or subcentimetric size (maximum diameters: 4 mm, mediastinal; 6 mm, para-aortic; 10 mm, right iliac), none meeting size criteria for pathological involvement. Whole-body bone scintigraphy (right) shows no skeletal metastases. Based on conventional imaging, the patient was staged as cN0 M0. (**B**) [^68^Ga]Ga-PSMA-11 PET/CT maximum intensity projection (MIP) reveals multiple PSMA-avid lymph nodes extending from the pelvis to the mediastinum, consistent with widespread nodal metastatic disease (miM1a). This case illustrates how PSMA PET/CT can detect molecularly defined metastatic disease beyond the reach of conventional size-based imaging criteria, leading to clinically meaningful stage migration and a fundamental change in treatment strategy—from curative-intent local therapy to systemic treatment.

In primary staging of prostate cancer, PSMA PET consistently outperforms conventional imaging, with multiple studies showing detection of lymph node metastases missed by standard modalities and consequent reclassification of disease stage in a substantial proportion of patients. Pooled analyses report upstaging rates of 20–30% or higher when PSMA PET is used instead of CT or MRI, particularly in intermediate- and high-risk populations, underscoring its clinical impact beyond diagnostic performance metrics [46]. A large multi-institutional European Association of Urology (EAU) further supports this real-world effect Young Academic Urologists study in high-risk cN0M0 patients, which demonstrated superior preoperative staging with PSMA PET and clinically meaningful stage migration prior to surgery (see an emblematic example in Figure 2) [47]. Less commonly, PSMA PET may also result in downstaging by clarifying equivocal nodal findings on conventional imaging. These advantages were confirmed in the randomized proPSMA trial, in which first-line PSMA PET/CT achieved higher staging accuracy and led to more frequent management changes compared with standard imaging [11].

The clinical impact of PSMA PET extends beyond diagnostic accuracy to treatment planning. The clinical value of PSMA PET lies less in its ability to replace histopathologic staging and more in its capacity to refine risk-adapted decision-making at critical therapeutic junctures. In surgical candidates, emerging evidence suggests that PSMA PET, particularly when integrated with clinical nomograms, may help refine selection for extended pelvic lymph node dissection in intermediate-risk patients, potentially reducing unnecessary dissections while acknowledging that microscopic nodal disease may still be missed [31,48,49]. By contrast, in high-risk patients, negative PSMA PET findings should be interpreted more cautiously, as occult micrometastatic nodal disease may still be present and may influence decisions regarding the extent or omission of ePLND. Recent efforts have focused on developing nomograms specifically adapted to PSMA PET findings. The Amsterdam–Brisbane–Sydney nomogram integrates lymph node status on PSMA PET with conventional clinicopathological variables, achieving an AUC of 83% (95%CI 0.78–0.88) [50]. Similarly, the updated Briganti 2023 nomogram, applied in PSMA PET N0 patients, achieved an AUC of 81% [51].

In radiotherapy planning, PSMA PET frequently influences target volume definition by identifying PET-positive lymph nodes. This can lead to both expansion of nodal target volumes and PET-guided dose escalation, but also recognition of “skip metastases” outside conventional pelvic nodal templates. In a large PSMA-PET/CT-based lymph node atlas of patients with nodal recurrence after radical prostatectomy, complete coverage by a standard RTOG clinical target volume was achieved in only 31.0% of lymph node metastases, while 51.8% were entirely outside, most notably located in the para-aortic, pararectal, paravesical, preacetabular, presacral, and inguinal regions, illustrating how PSMA PET may reveal disease beyond expected drainage areas and thereby alter CTV delineation [52]. More broadly, a recent review emphasized that PSMA PET has had a substantial impact on radiation oncology planning across both primary and recurrent settings by improving identification of nodal disease and enabling individualized field design and simultaneous integrated boosts to PET-positive lesions [53]. A recent clinical planning study demonstrated that [^68^Ga]Ga-PSMA PET/CT altered the planned planning target volume in a substantial proportion of patients undergoing both salvage and definitive radiotherapy [54]. In the salvage setting, early outcome data suggest promising results, with PSMA PET-guided elective nodal radiotherapy achieving 3-year biochemical recurrence-free survival rates of approximately 72% [55].

In the setting of biochemical recurrence, the clinical value of PSMA PET lies in its ability to achieve clinically meaningful detection rates even at low PSA levels and to localize disease as nodal-only recurrence, directly informing salvage treatment strategies. In a large prospective trial [^68^Ga]Ga-PSMA-11 PET demonstrated clinically meaningful detection rates even at low PSA levels (e.g., approximately 38% for PSA levels <0.5 ng/mL), with detection rates increasing alongside PSA [56]. Detection of occult nodal or distant metastases on PSMA PET/CT may prompt consideration of earlier systemic therapy, salvage lymph-node directed therapies or treatment intensification [57]. However, despite its high specificity, sensitivity remains limited for the molecularly defined metastatic population, and a negative PSMA PET scan does not exclude nodal involvement [58,59]. Prospective randomized studies incorporating PSMA PET as the primary staging modality are therefore needed to clarify the therapeutic implications of PSMA-detected disease.

Both the EAU and the American Urological Association (AUA) guidelines reflect this evolving evidence base. The EAU recommends PSMA PET/CT, when available, for staging in high-risk localized and locally advanced prostate cancer because of its superior detection of nodal and distant metastases compared with conventional imaging and suggests its use in selected intermediate-risk patients to refine staging. The EAU also notes that PSMA PET has altered management in a substantial proportion of patients in prospective studies, while emphasizing that outcome data based solely on PET-guided decisions are still maturing [3]. This ongoing guideline caution reflects not uncertainty about the diagnostic accuracy of PSMA PET, but persistent ambiguity regarding how molecularly detected nodal disease should be weighted relative to established clinical and pathological risk factors. Similarly, the AUA endorses PSMA PET, when available, as the preferred advanced imaging modality in patients with suspected metastatic disease prior to definitive local therapy, acknowledging that PSMA PET frequently identifies nodal disease not detected by CT or MRI and may meaningfully influence treatment planning [60].

Importantly, PSMA PET facilitates treatment personalization by enabling escalation in patients with molecularly identified nodal disease, while potentially reducing overtreatment in those without PET-detectable disease and avoiding undertreatment in patients whose true nodal burden would be underestimated by conventional imaging alone [11,38,61]. However, management change should not be equated with proven clinical benefit. Although PSMA PET frequently alters staging and treatment planning, robust prospective evidence demonstrating that PSMA PET-guided treatment adaptation improves long-term oncologic outcomes remains limited. Guideline committees emphasize that PSMA PET findings must be interpreted in the context of overall clinical risk, recognizing that microscopic nodal disease below the resolution of PET imaging may still be present [2,3,16].

### 2.5. Limitations, Pitfalls, and Controversies in Lymph Node Assessment

Nonetheless, PSMA PET has several clinically relevant limitations in the assessment of lymph node metastases (Table 2). False-positive findings represent a well-recognized pitfall and may arise from multiple mechanisms. First, reactive or inflammatory lymph nodes can demonstrate low to moderate PSMA uptake, reflecting increased vascularity and immune activity rather than malignant infiltration [62]. Similarly, several physiologic structures, most notably the sympathetic ganglia, including the stellate, celiac, and sacral ganglia, can also be misinterpreted as nodal metastases if not carefully correlated with anatomical imaging [62]. In addition, unspecific PSMA uptake may occur in a variety of benign conditions, particularly those associated with increased bone turnover or neovascularization [63,64]. Degenerative bone disease, healing fractures, and other reactive skeletal processes are common sources of uptake and may lead to false-positive skeletal findings, especially when using ^18^F-labeled tracers such as [^18^F]PSMA-1007,which have been associated with a higher incidence of nonspecific skeletal uptake [63,65]. Importantly, PSMA expression is also not entirely specific to prostate cancer, as it is also present in the neovasculature of various non-prostatic malignancies and in certain benign lesions. As a result, uptake may be observed in tumors such as renal cell carcinoma, hepatocellular carcinoma, and lung cancer, as well as in benign entities including hemangiomas and granulomatous diseases. These findings may mimic nodal or distant metastatic disease and increase if not interpreted in the appropriate clinical and imaging context [63,66]. Awareness of these potential pitfalls is essential for accurate interpretation.

Conversely, false-negative findings remain a critical limitation. PSMA PET has reduced sensitivity for micrometastatic nodal disease, as small-volume metastases frequently fall below the spatial resolution of PET imaging. However, beyond these technical constraints, biological factors play an equally important role. PSMA expression is inherently heterogeneous, both within individual tumors and across metastatic sites, and a subset of prostate cancers may exhibit low or absent PSMA expression [67]. This heterogeneity is dynamic and may evolve over the course of disease progression or treatment, particularly in advanced disease states characterized by lineage plasticity, such as neuroendocrine differentiation of androgen receptor-independent phenotypes, are frequently associated with downregulation or loss of PSMA expression [20], As a result, lymph node metastases that are biologically aggressive or dedifferentiated may not be visualized on PSMA PET despite being clinically significant. This limitation is further compounded by the inability of PET imaging to resolve intralesional heterogeneity below its spatial resolution, meaning that PSMA-negative tumor components may remain undetected even within otherwise PSMA-avid disease. Consequently, a negative PSMA PET scan does not reliably exclude lymph node involvement, especially in patients with high-risk or biologically aggressive disease [31,68]. This limitation is particularly relevant in patients with a high pretest probability of nodal involvement, in whom occult metastases may still be identified at extended pelvic lymph node dissection despite negative PSMA PET findings, reinforcing the continued role of surgical staging in selected high-risk cases.

These considerations underscore the importance of integrating PSMA PET findings with conventional imaging, clinical risk stratification, and, where appropriate, histopathological confirmation. In addition, given the documented interobserver variability in PSMA-PET interpretation and the demonstrated improvement in staging accuracy in centers with active multidisciplinary teams, integration of molecular imaging findings into structured multidisciplinary discussions should be considered essential to mitigate misclassification and avoid inappropriate treatment escalation [25,69].

Another important challenge is the lack of fully standardized interpretation criteria for nodal disease. Although structured reporting systems such as PROMISE and PSMA-RADS have improved consistency, they are not uniformly adopted in routine clinical practice [70,71]. This contributes to inter-reader and inter-center variability, particularly in cases with low-level tracer uptake or borderline lymph node findings and may limit reproducibility across institutions. In addition, access to PSMA PET varies globally, limited radiotracer availability, regulatory constraints and their implementation in routine practice remains heterogeneous, contributing to ongoing uncertainty about the most effective and evidence-based integration of PSMA PET into lymph node staging relative to surgical and radiotherapeutic strategies [62,72,73].

From a health economics perspective, the integration of PSMA PET into clinical decision-making must be balanced against its higher upfront costs. Emerging evidence suggests that PSMA PET may achieve a favorable cost–benefit profile in selected clinical scenarios by reducing unnecessary invasive procedures and improving diagnostic accuracy. For example, a recent cost-effectiveness analysis demonstrated that incorporating PSMA PET/CT into the diagnostic pathway for equivocal MRI findings (PI-RADS 3) can improve quality-adjusted life years (QALYs) while remaining borderline cost-effective, partly by avoiding unnecessary biopsies and reducing overdiagnosis of low-risk disease [74]. More broadly, systematic evaluations indicate that PSMA PET is generally cost-effective compared with conventional imaging across multiple healthcare settings, although results vary depending on local resource availability and clinical context [74].

Collectively, these limitations underpin ongoing debate regarding the optimal role of PSMA PET in clinical decision-making and reinforce the importance of interpreting imaging findings within a comprehensive clinical, pathological, and risk-adapted framework.

### 2.6. Comparison with Other Emerging Imaging Techniques

Before the advent of PSMA PET, choline PET/CT was widely used for staging and restaging prostate cancer; however, its role has diminished as studies have demonstrated superior lesion detectability with PSMA-targeted tracers, particularly for lymph node metastases and at lower disease burden [75,76]. Whole-body MRI (WB-MRI) remains an attractive radiation-free alternative and performs well for skeletal disease, but comparative data in primary nodal staging suggest that PSMA PET provides greater diagnostic confidence for lymph node assessment, although both modalities may miss micrometastatic disease [73,77]. Hybrid PSMA PET/MRI seeks to combine the molecular sensitivity of PET with the superior soft-tissue contrast of MRI. Systematic reviews indicate that this approach may offer advantages for local and regional evaluation, including nodal and intraprostatic staging, while providing broadly comparable performance to PET/CT for detecting distant metastases, albeit with reduced availability and increased technical complexity [78,79].

Beyond visual interpretation, radiomics and artificial intelligence approaches are being explored to extract quantitative features from PSMA PET that may improve reproducibility, automate lesion detection, including lymph nodes, and enhance risk stratification. Radiomic analyses have shown promise in correlating imaging features with tumor aggressiveness, lymph node involvement, and biochemical recurrence. AI-driven models may also help address diagnostic challenges, such as differentiating physiological uptake (e.g., ganglia) from true nodal metastases, and have demonstrated improved accuracy compared with visual assessment in some studies. However, current evidence remains heterogeneous and retrospective, and these tools should be regarded as investigational adjuncts rather than practice-changing solutions at present [80,81].

Finally, alternative molecular targets are under investigation for PSMA-negative or biologically distinct disease. ^18^F-fluciclovine has been studied extensively (especially in recurrence and in some primary-staging contexts), while GRPR-targeted tracers (e.g., ^68^Ga-RM2) represent a promising complementary pathway. Early clinical studies suggest potential utility in selected patients, although these agents remain less validated than PSMA PET for routine lymph node staging [82,83,84].

## 3. Future Perspectives and Research Directions

A key unresolved question is whether PSMA PET can meaningfully replace ePLND in primary staging. An important limitation of the current evidence base is its heterogeneity. Although high-quality prospective studies support the diagnostic superiority of PSMA PET over conventional imaging, much of the evidence regarding treatment adaptation, real-world implementation, radiotherapy planning, and surgical de-escalation remains derived from retrospective series, registry analyses, surveys, or expert consensus. Despite consistently high specificity, prospective studies validated against histopathology demonstrate limited sensitivity, approximately 40%, for detecting pelvic nodal metastases, indicating that a negative PSMA PET scan cannot reliably exclude micrometastatic disease. On this basis, current evidence does not support PSMA PET as a standalone alternative to ePLND in surgical candidates [45]. A more realistic near-term approach is risk-adapted management, with selective de-escalation through omission or tailoring of ePLND in carefully chosen lower-risk patients with negative PSMA PET, and targeted escalation when PET identifies nodal disease likely to alter management, strategies that require prospective validation.

Such validation is now underway, with randomized and multicenter trials designed to determine whether PSMA PET-guided planning improves clinically meaningful outcomes rather than simply refining staging. One example is the phase III PSMA dRT trial (NCT04457245), which evaluates PSMA PET/CT-guided planning prior to definitive radiotherapy in unfavorable intermediate- and high-risk patients to assess whether PET-driven treatment modifications translate into improved clinical endpoints [65]. Beyond traditional staging paradigms, the field is increasingly moving toward imaging-guided and biology-driven strategies. PSMA PET is being used to direct focal treatment to PET-positive lymph nodes through dose escalation or metastasis-directed therapy, and to integrate molecular imaging findings with baseline clinical risk models to better personalize treatment intensity. Standardized frameworks such as miTNM (PROMISE) and its updated PROMISE V2 support this approach by promoting structured reporting that captures not only the location and extent of nodal disease, but also semi-quantitative measures of PSMA expression, thereby helping to link imaging phenotype with therapeutic decision-making [14,85].

In parallel, PSMA-targeted technologies are increasingly being extended into the intraoperative setting through radio-guided surgery. PSMA-targeted radio-guided surgery (PSMA-RGS) combines preoperative molecular imaging with intraoperative γ-probe detection, allowing real-time localization of PSMA-avid lesions and facilitating more precise identification and resection of metastatic lymph nodes [86]. A recent systematic review and meta-analysis by Wen et al. (2026) reported high diagnostic accuracy across multiple anatomical levels, supporting the feasibility of this approach as an adjunct to surgical staging [87]. This technique may be particularly valuable for detecting small or anatomically challenging nodal metastases that are not easily identified during conventional surgery, potentially improving surgical completeness. Furthermore, the development of hybrid tracers integrating radio guidance with fluorescence imaging represents an additional step toward enhancing intraoperative visualization [88]. Lastly, accurate characterization of PSMA-positive nodal disease has increasing therapeutic relevance in metastatic castration-resistant prostate cancer. Trials such as TheraP and PSMAfore demonstrate that PSMA PET is not only a staging tool, but also a biomarker for treatment eligibility and patient selection for [^177^Lu]Lu-PSMA-617 [89,90,91]. Although these approaches remain investigational and are not yet incorporated into routine clinical practice, they exemplify the evolving role of PSMA-targeted strategies in bridging imaging and surgical intervention, with future studies needed to assess their impact on long-term oncologic outcomes.

More broadly, PSMA PET-based approaches are contributing to a shift toward molecularly defined staging, in which lymph node classification reflects not only anatomic burden but also target expression and tumor biology. This evolving paradigm may have direct implications for treatment selection and risk stratification, although further prospective studies are required to determine its impact on long-term oncologic outcomes 86.

Thus, to sum up, several clinically relevant questions remain unresolved and should be prioritized in future prospective studies:

- Can PSMA PET safely guide de-escalation of ePLND? Prospective studies are needed to define which patients, particularly in intermediate-risk and selected high-risk groups, can safely avoid or undergo a tailored lymph node dissection.
- Do PSMA PET-guided treatment changes improve oncologic outcomes? While PSMA PET frequently leads to stage migration and management changes, it remains unclear whether these adaptations translate into improved biochemical recurrence-free, metastasis-free, cancer-specific, or overall survival.
- How should PET-positive nodal disease alter treatment intensity? The optimal management of PSMA-detected pelvic nodal disease and extra-pelvic M1a disease remains uncertain, particularly regarding systemic therapy, elective nodal radiotherapy, focal dose escalation, and metastasis-directed treatment.
- How should negative scans be interpreted in high-risk patients? Because micrometastatic nodal disease may remain below PET resolution, future work should clarify how PSMA PET should be combined with nomograms, MRI, PSA kinetics, biopsy grade, and other clinical risk factors.
- How can interpretation and reporting be standardized? Wider validation and adoption of structured frameworks such as PROMISE/miTNM and PSMA-RADS are needed to reduce inter-reader variability and improve reproducibility across centers.
- What is the optimal tracer and imaging protocol for nodal staging? Comparative studies should determine whether tracer selection, delayed imaging, diuretic protocols, or acquisition parameters meaningfully improve nodal detection and clinical decision-making.
- Can PSMA-targeted radio-guided surgery improve outcomes? PSMA-radio-guided and hybrid radiofluorescence-guided approaches are promising, but their impact on surgical completeness, morbidity, recurrence patterns, and long-term survival requires prospective validation.
- How should cost, access, and implementation be addressed? Future research should assess how PSMA PET can be integrated into routine care across different healthcare systems, considering radiotracer availability, reimbursement, infrastructure, and clinical impact.

## 4. Conclusions

PSMA PET has markedly improved lymph node staging in prostate cancer by allowing disease detection at a molecular level, beyond what conventional imaging can achieve. It should be viewed not as a replacement for existing nodal staging paradigms, but as a complementary tool that reshapes how nodal disease is conceptualized and operationalized in contemporary prostate cancer care. While its high specificity often leads to meaningful changes in management, limited sensitivity for micrometastatic disease means it cannot replace extended pelvic lymph node dissection as a standalone staging tool. Instead, its greatest strength lies in supporting risk-adapted treatment decisions. Ongoing clinical trials, greater standardization, and closer integration with established risk models will determine how PSMA PET is best used to guide nodal management.

## Figures and Tables

**Table 1:** Comparison of commonly used PSMA radiotracers for lymph node staging.

Radiotracer	Production	Half-Life	Excretion Pattern	Key Advantages	Limitations	Clinical Implications for Lymph Node Staging
**[^68^Ga]Ga-PSMA-11**	Generator-based, on-site production	~68 min	High, predominantly renal	Widely validated; high specificity (~95%); strong clinical evidence	Urinary activity may obscure pelvic nodes; limited sensitivity for micro metastases	Standard reference tracer; reliable but limited near bladder/prostatic bed
**[^18^F]PSMA-1007**	Cyclotron-based, centralized production	~110–120 min	Mainly (hepatobiliary)	Low urinary activity improves pelvic evaluation; longer half-life allows distribution	Higher nonspecific uptake, especially bone; possible false positives	Advantageous for pelvic nodal assessment near bladder/ureters; requires cautious interpretation
**[^18^F]rhPSMA-7.3**	Cyclotron-based production	~110 min	Reduced (vs. [^68^Ga]Ga-PSMA-11)	FDA-approved; Sp 93–97%; reduced bladder activity	Urinary activity may affect pelvic assessment	Favorable pelvic assessment; NCCN-listed; Phase 3 data
**[^18^F]DCFPyL**	Cyclotron-based, centralized production	~110–120 min	Moderate (rapid clearance)	High specificity (~96–99%); good image quality; scalable production	Urinary activity may affect pelvic assessment; no clear superiority over other tracers	Comparable to Ga-PSMA-11; slightly improved pelvic assessment vs. Ga tracers
**[^18^F]JK-PSMA-7**	Cyclotron-based production	~110 min	Mixed (renal + hepatobiliary)	High TBR; equivalent to ^68^Ga-PSMA-11; growing EU experience	No proven superiority over other tracers	Dual excretion may reduce bladder interference

**Table 2:** Main limitations and pitfalls of PSMA PET in lymph node assessment: clinical examples.

Limitation/Pitfall	Mechanism	Clinical Example
**False-positive lymph node uptake**	Reactive or inflammatory nodes may show low-to-moderate PSMA uptake due to increased vascularity or immune activation.	Mild PSMA uptake in pelvic or inguinal nodes in a patient with recent infection, inflammation, or post-procedural change.
**Physiological ganglia uptake**	Sympathetic ganglia can show PSMA uptake and mimic nodal metastases.	Sacral, stellate, or celiac ganglia mistaken for nodal disease, particularly when uptake is elongated or located along typical ganglion anatomy.
**Benign bone or soft-tissue uptake**	Increased bone turnover, trauma, degenerative disease, or benign vascular lesions may show nonspecific uptake.	Rib uptake from a healing fracture, degenerative spinal uptake, hemangioma, or granulomatous lesion misread as metastatic disease.
**Tracer-specific nonspecific uptake**	Some tracers may show more nonspecific skeletal uptake.	Isolated low-level bone uptake without CT correlate in a patient otherwise staged for nodal disease.
**Limited sensitivity for micrometastatic nodal disease**	Small-volume nodal metastases may fall below PET spatial resolution.	Negative PSMA PET in a high-risk patient who later has microscopic nodal metastases at ePLND.
**PSMA expression heterogeneity**	PSMA expression varies within and between lesions; dedifferentiated or neuroendocrine-like tumors may have low/absent PSMA expression.	Aggressive disease with discordantly low PSMA uptake despite clinical suspicion or rising PSA.
**Urinary tracer activity obscuring pelvic disease**	Renal excretion and bladder activity may obscure small pelvic nodes or prostate-bed recurrence, particularly with [^68^Ga] Ga-PSMA-11.	Small obturator/internal iliac node or prostate-bed lesion hidden by urinary activity.
**Inter-reader and inter-center variability**	PROMISE/miTNM and PSMA-RADS improve consistency but are not universally adopted.	Borderline low-level nodal uptake classified differently across centers.
**Limited outcome-based validation**	Most therapeutic trials were designed around conventional imaging, not PSMA PET-defined disease states.	PET-detected M1a nodal disease leading to systemic treatment intensification despite limited long-term data for that exact pathway.
**Access, cost, and availability issues**	Radiotracer production, regulatory approval, infrastructure, and reimbursement vary by region.	A patient eligible for PSMA PET according to guidelines but unable to access imaging in a timely manner.

## Data Availability

No new data were created or analyzed in this study.

## References

[1] Karwacki J., Mioskowska A., Tomecka P., Mączka K., Gurwin A., Kobylański M., Hałoń A., Szuba P., Zdrojowy R., Szydełko T. (2025). Metastatic lymph nodes outside the extended lymphadenectomy template correlate with advanced staging but not grading in prostate cancer patients undergoing radical prostatectomy. Int. Urol. Nephrol..

[2] Cornford P., van den Bergh R.C.N., Briers E., Van den Broeck T., Brunckhorst O., Darraugh J., Eberli D., De Meerleer G., De Santis M., Farolfi A. (2024). EAU-EANM-ESTRO-ESUR-ISUP-SIOG Guidelines on Prostate Cancer—2024 Update. Part I: Screening, Diagnosis, and Local Treatment with Curative Intent. Eur. Urol..

[3] Tilki D., van den Bergh R.C.N., Briers E., Van den Broeck T., Brunckhorst O., Darraugh J., Eberli D., De Meerleer G., De Santis M., Farolfi A. (2024). EAU-EANM-ESTRO-ESUR-ISUP-SIOG Guidelines on Prostate Cancer. Part II—2024 Update: Treatment of Relapsing and Metastatic Prostate Cancer. Eur. Urol..

[4] Aluwini S., Oprea-Lager D.E., de Barros H., Mehra N., Stoevelaar H., Yakar D., van der Poel H., Dutch M1a Prostate Cancer Working Group (2021). M1a prostate cancer: Results of a Dutch multidisciplinary consensus meeting. BJUI Compass.

[5] Ghafoor S., Burger I.A., Vargas A.H. (2019). Multimodality Imaging of Prostate Cancer. J. Nucl. Med..

[6] Gandaglia G., Ploussard G., Valerio M., Mattei A., Fiori C., Fossati N., Stabile A., Beauval J.-B., Malavaud B., Roumiguié M. (2019). A Novel Nomogram to Identify Candidates for Extended Pelvic Lymph Node Dissection Among Patients with Clinically Localized Prostate Cancer Diagnosed with Magnetic Resonance Imaging-targeted and Systematic Biopsies. Eur. Urol..

[7] Memorial Sloan Kettering Cancer Center Prostate Cancer Nomograms: Pre-Radical Prostatectomy. https://www.mskcc.org/nomograms/prostate/pre_op.

[8] Małkiewicz B., Kiełb P., Karwacki J., Czerwińska R., Długosz P., Lemiński A., Nowak Ł., Krajewski W., Szydełko T. (2022). Utility of Lymphadenectomy in Prostate Cancer: Where Do We Stand?. J. Clin. Med..

[9] Kong J., Lichtbroun B., Sterling J., Wang Y., Wang Q., Singer E.A., Jang T.L., Ghodoussipour S., Kim I.Y. (2022). Comparison of perioperative complications for extended vs standard pelvic lymph node dissection in patients undergoing radical prostatectomy for prostate cancer: A meta-analysis. Am. J. Clin. Exp. Urol..

[10] von Stauffenberg F., Poyet C., Beintner-Skawran S., Maurer A., Schmid F.A. (2024). Current Clinical Applications of PSMA-PET for Prostate Cancer Diagnosis, Staging, and Treatment. Cancers.

[11] Hofman M.S., Lawrentschuk N., Francis R.J., Tang C., Vela I., Thomas P., Rutherford N., Martin J.M., Frydenberg M., Shakher R. (2020). Prostate-specific membrane antigen PET-CT in patients with high-risk prostate cancer before curative-intent surgery or radiotherapy (proPSMA): A prospective, randomised, multicentre study. Lancet.

[12] Hofman M.S., Kasivisvanathan V., Link E., Buteau J., Roberts M.J., Francis R.J., Tang C., Vela I., Thomas P., Rutherford N. (2024). Baseline Nodal Status on ^68^Ga-PSMA-11 Positron Emission Tomography/Computed Tomography in Men with Intermediate- to High-risk Prostate Cancer Is Prognostic for Treatment Failure: Follow-up of the proPSMA Trial. Eur. Urol. Oncol..

[13] Djaïleb L., Armstrong W.R., Thompson D., Gafita A., Farolfi A., Rajagopal A., Grogan T.R., Nguyen K., Benz M.R., Hotta M. (2023). Presurgical ^68^Ga-PSMA-11 Positron Emission Tomography for Biochemical Recurrence Risk Assessment: A Follow-up Analysis of a Multicenter Prospective Phase 3 Imaging Trial. Eur. Urol..

[14] Karpinski M.J., Hüsing J., Claassen K., Möller L., Kajüter H., Oesterling F., Grünwald V., Umutlu L., Kleesiek J., Telli T. (2024). Combining PSMA-PET and PROMISE to re-define disease stage and risk in patients with prostate cancer: A multicentre retrospective study. Lancet Oncol..

[15] Karpinski M.J., Rahbar K., Bögemann M., Nikoukar L.R., Schäfers M., Hoberück S., Miederer M., Hölscher T., Rasul S., Miszczyk M. (2025). Updated Prostate Cancer Risk Groups by Prostate-specific Membrane Antigen Positron Emission Tomography Prostate Cancer Molecular Imaging Standardized Evaluation (PPP2): Results from an International Multicentre Registry Study. Eur. Urol..

[16] Schaeffer E.M., Srinivas S., Adra N., An Y., Barocas D., Bitting R., Bryce A., Chapin B., Cheng H.H., D’aMico A.V. (2023). Prostate Cancer, Version 4.2023, NCCN Clinical Practice Guidelines in Oncology. J. Natl. Compr. Cancer Netw..

[17] Fizazi K., Gillessen S. (2023). Updated treatment recommendations for prostate cancer from the ESMO Clinical Practice Guideline considering treatment intensification and use of novel systemic agents. Ann. Oncol..

[18] Matrone F., Urso L., Girometti R., Polesel J., Sepulcri M., Pierantoni F., Artioli P., Caliò A., Campo I., Cimadamore A. (2025). The expanding role of next-generation imaging in prostate cancer management: A cross-sectional survey exploring the clinical practice of uro-oncologists in North-Eastern Italy; on behalf of GUONE (Gruppo Uro-Oncologico del Nord-Est). Ther. Adv. Urol..

[19] Bauckneht M., Evangelista L., Sofia L., Maccauro M., Filice A., De Rimini M.L., Caffo O., Messina C., Maruzzo M., Pinterpe G. (2025). Joint survey by AIMN, AIOM, AIRO, SIU, SIUrO, and Meet-URO about the use of PSMA PET imaging in prostate cancer in Italy: Technical aspects and primary staging setting. Clin. Transl. Imaging.

[20] Bakht M.K., Beltran H. (2025). Biological determinants of PSMA expression, regulation and heterogeneity in prostate cancer. Nat. Rev. Urol..

[21] Sandhu K., Chen D., Hennes D., Murphy D.G., Lawrentschuk N., Perera M. (2025). PSMA Theranostics in Prostate Cancer and Beyond: Current and Future Perspectives. Cancers.

[22] Maes J., Gesquière S., De Spiegeleer A., Maes A., Van de Wiele C. (2024). Prostate-Specific Membrane Antigen Biology and Pathophysiology in Prostate Carcinoma, an Update: Potential Implications for Targeted Imaging and Therapy. Int. J. Mol. Sci..

[23] Naik M., Khan S.R., Lewington V., Challapalli A., Eccles A., Barwick T.D. (2024). Imaging and therapy in prostate cancer using prostate specific membrane antigen radioligands. Br. J. Radiol..

[24] Schwarzenboeck S.M., Rauscher I., Bluemel C., Fendler W.P., Rowe S.P., Pomper M.G., Asfhar-Oromieh A., Herrmann K., Eiber M. (2017). PSMA Ligands for PET Imaging of Prostate Cancer. J. Nucl. Med..

[25] Wang L., Wang L., Wang X., Wu D. (2025). The Evolving Role of PSMA-PET/CT in Prostate Cancer Management: An Umbrella Review of Diagnostic Restaging, Therapeutic Redirection, and Survival Impact. Curr. Oncol. Rep..

[26] Huang S., Ong S., McKenzie D., Mirabelli A., Chen D.C., Chengodu T., Murphy D.G., Hofman M.S., Lawrentschuk N., Perera M. (2024). Comparison of ^18^F-based PSMA radiotracers with [^68^Ga]Ga-PSMA-11 in PET/CT imaging of prostate cancer—A systematic review and meta-analysis. Prostate Cancer Prostatic Dis..

[27] Fendler W.P., Eiber M., Beheshti M., Bomanji J., Calais J., Ceci F., Cho S.Y., Fanti S., Giesel F.L., Goffin K. (2023). PSMA PET/CT: Joint EANM procedure guideline/SNMMI procedure standard for prostate cancer imaging 2.0. Eur. J. Nucl. Med. Mol. Imaging.

[28] Vyas M., Henderson A.M., Leslie S.W. (2026). Nuclear Medicine Applications in Prostate Cancer. StatPearls [Internet].

[29] Sadaghiani M.S. (2022). Pelvic Nodal Metastasis Detection with ^68^Ga-PSMA-11 PET Prior to Radical Prostatectomy and Pelvic Lymph Node Dissection. Radiol. Imaging Cancer.

[30] Kopp J., Kopp D., Bernhardt E., Manka L., Beck A., Gerullis H., Karakiewicz P., Schoerner W., Hammerer P., Schiffmann J. (2020). ^68^Ga-PSMA PET/CT based primary staging and histological correlation after extended pelvic lymph node dissection at radical prostatectomy. World J. Urol..

[31] Hope T.A., Eiber M., Armstrong W.R., Juarez R., Murthy V., Lawhn-Heath C., Behr S.C., Zhang L., Barbato F., Ceci F. (2021). Diagnostic Accuracy of ^68^Ga-PSMA-11 PET for Pelvic Nodal Metastasis Detection Prior to Radical Prostatectomy and Pelvic Lymph Node Dissection. JAMA Oncol..

[32] Bauckneht M., Miceli A., Signori A., Albano D., Capitanio S., Piva R., Laudicella R., Franchini A., D’aMico F., Riondato M. (2023). Combined forced diuresis and late acquisition on [^68^Ga]Ga-PSMA-11 PET/CT for biochemical recurrent prostate cancer: A clinical practice-oriented study. Eur. Radiol..

[33] Dullea A., O’SUllivan L., Carrigan M., Ahern S., McGarry M., O’BRien K., Harrington P., Walsh K.A., Smith S.M., Ryan M. (2025). Diagnostic accuracy of ^18^F Prostate Specific Membrane Antigen (PSMA) PET-CT radiotracers in staging and restaging of high-risk prostate cancer patients and patients with biochemical recurrence: Protocol for an overview of reviews. HRB Open Res..

[34] Rauscher I., Kroenke M., König M., Gafita A., Maurer T., Horn T., Schiller K., Weber W., Eiber M. (2020). Matched-Pair Comparison of ^68^Ga-PSMA-11 PET/CT and ^18^F-PSMA-1007 PET/CT: Frequency of Pitfalls and Detection Efficacy in Biochemical Recurrence After Radical Prostatectomy. J. Nucl. Med..

[35] Surasi D.S., Eiber M., Maurer T., Preston M.A., Helfand B.T., Josephson D., Tewari A.K., Somford D.M., Rais-Bahrami S., Koontz B.F. (2023). Diagnostic Performance and Safety of Positron Emission Tomography with ^18^F-rhPSMA-7.3 in Patients with Newly Diagnosed Unfavourable Intermediate- to Very-high-risk Prostate Cancer: Results from a Phase 3, Prospective, Multicentre Study (LIGHTHOUSE). Eur. Urol..

[36] Jani A.B., Ravizzini G.C., Gartrell B.A., Siegel B.A., Twardowski P., Saltzstein D., Fleming M.T., Chau A., Davis P., Chapin B.F. (2023). Diagnostic Performance and Safety of ^18^ F-rhPSMA-7.3 Positron Emission Tomography in Men with Suspected Prostate Cancer Recurrence: Results from a Phase 3, Prospective, Multicenter Study (SPOTLIGHT). Reply. J. Urol..

[37] Spratt D.E., Srinivas S., Adra N., Ahmed B., An Y., Bitting R., Chapin B., Cheng H.H., Cho S.Y., D’aMico A.V. (2025). Prostate Cancer, Version 3.2026, NCCN Clinical Practice Guidelines in Oncology. J. Natl. Compr. Cancer Netw..

[38] Stabile A., Pellegrino A., Mazzone E., Cannoletta D., de Angelis M., Barletta F., Scuderi S., Cucchiara V., Gandaglia G., Raggi D. (2022). Can Negative Prostate-specific Membrane Antigen Positron Emission Tomography/Computed Tomography Avoid the Need for Pelvic Lymph Node Dissection in Newly Diagnosed Prostate Cancer Patients? A Systematic Review and Meta-analysis with Backup Histology as Reference Standard. Eur. Urol. Oncol..

[39] Chong B., Khor V., Hoo J.X., Lee A., Tan Y.G., Ho H., Cheng C., Tay K.J., Tuan J., Yuen J. (2025). Pelvic lymph node management in prostate cancer: A narrative review. Prostate Int..

[40] Incesu R.-B., Preisser F., Pompe R.S., Nohe F., Mandel P., Maurer T., Graefen M., Tilki D. (2025). Diagnostic Accuracy of Prostate-specific Membrane Antigen Positron Emission Tomography/Computed Tomography for Primary Lymph Node Staging Before Radical Prostatectomy. Eur. Urol. Focus.

[41] Yang B., Dong H., Zhang S., Ming S., Yang R., Peng Y., Gao X. (2025). PSMA PET vs. mpMRI for Lymph Node Metastasis of Prostate Cancer: A Systematic Review and Head-to-Head Comparative Meta-analysis. Acad. Radiol..

[42] Mazzone E., Cannoletta D., Quarta L., Chen D.C., Thomson A., Barletta F., Stabile A., Moon D., Eapen R., Lawrentschuk N. (2025). A Comprehensive Systematic Review and Meta-analysis of the Role of Prostate-specific Membrane Antigen Positron Emission Tomography for Prostate Cancer Diagnosis and Primary Staging before Definitive Treatment. Eur. Urol..

[43] Maestroni U.V., Campobasso D., Guarino G., Acampora A., Scarlattei M., Ziglioli F., Dinale F., Baldari G., Migliari S., Gasparro D. (2023). Lymph node staging with ^68^Ga-PSMA PET in patients with intermediate and high-risk prostate cancer suitable for radical prostatectomy managed in a prostate cancer unit. Chin. Clin. Oncol..

[44] Shen Z., Li Z., Li Y., Tang X., Lu J., Chen L., Cheng Z.Z., Liao H., Zhou S. (2025). PSMA PET/CT for prostate cancer diagnosis: Current applications and future directions. J. Cancer Res. Clin. Oncol..

[45] Jansen B.H.E., Bodar Y.J.L., Zwezerijnen G.J.C., Meijer D., van der Voorn J.P., Nieuwenhuijzen J.A., Wondergem M., Roeleveld T.A., Boellaard R., Hoekstra O.S. (2021). Pelvic lymph-node staging with ^18^F-DCFPyL PET/CT prior to extended pelvic lymph-node dissection in primary prostate cancer—The SALT trial-. Eur. J. Nucl. Med. Mol. Imaging.

[46] Jeet V., Parkinson B., Song R., Sharma R., Hoyle M. (2023). Histopathologically Validated Diagnostic Accuracy of PSMA-PET/CT in the Primary and Secondary Staging of Prostate Cancer and the Impact of PSMA-PET/CT on Clinical Management: A Systematic Review and Meta-analysis. Semin. Nucl. Med..

[47] Bianchi L., Droghetti M., Catanzaro C., Valerio M., Novello Q., Mari A., Campi R., Nicoletti R., Heidegger I., Giannini G. (2025). Prostate-specific Membrane Antigen Positron Emission Tomography Versus Conventional Imaging for Preoperative Staging High-risk Prostate Cancer Patients Undergoing Surgery for cN0M0 Disease: An European Association of Urology—Young Academic Urologists Prostate Cancer Working Group Multi-institutional Study. Eur. Urol. Oncol..

[48] Bauckneht M., Checcucci E., Lanfranchi F., Cisero E., Rizzo A., Scuderi S., Barletta F., Robesti D., Amparore D., Sterrantino A. (2025). The added value of PSMA PET to nomograms in identifying optimal candidates for extended pelvic lymph node dissection in intermediate-risk prostate cancer patients. Minerva Urol. Nephrol..

[49] Incesu R.-B., Preisser F., Nohe F., Maurer T., Graefen M., Tilki D. (2025). Negative PSMA PET can be used to avoid unnecessary pelvic lymph node dissection in intermediate risk prostate cancer. Prostate Cancer Prostatic Dis..

[50] Gandaglia G., Barletta F., Robesti D., Scuderi S., Rajwa P., Rivas J.G., Ibanez L., Soeterik T.F., Bianchi L., Afferi L. (2023). Identification of the Optimal Candidates for Nodal Staging with Extended Pelvic Lymph Node Dissection Among Prostate Cancer Patients Who Underwent Preoperative Prostate-specific Membrane Antigen Positron Emission Tomography. External Validation of the Memorial Sloan Kettering Cancer Center and Briganti Nomograms and Development of a Novel Tool. Eur. Urol. Oncol..

[51] Vis A.N., Meijer D., Roberts M.J., Siriwardana A.R., Morton A., Yaxley J.W., Samaratunga H., Emmett L., van de Ven P.M., Heymans M.W. (2023). Development and External Validation of a Novel Nomogram to Predict the Probability of Pelvic Lymph-node Metastases in Prostate Cancer Patients Using Magnetic Resonance Imaging and Molecular Imaging with Prostate-specific Membrane Antigen Positron Emission Tomography. Eur. Urol. Oncol..

[52] Schiller K., Stöhrer L., Düsberg M., Borm K., Devecka M., Vogel M.M., Tauber R., Heck M.M., Rauscher I., Eiber M. (2021). PSMA-PET/CT–based Lymph Node Atlas for Prostate Cancer Patients Recurring After Primary Treatment: Clinical Implications for Salvage Radiation Therapy. Eur. Urol. Oncol..

[53] Spohn S.K., Grosu A.-L. (2025). Impact of PSMA PET on Radiation Oncology Planning. Semin. Nucl. Med..

[54] Bock F., Frerker B., Schubert L., Rennau H., Kurth J., Krause B.J., Hildebrandt G., Schwarzenböck S.M. (2024). Impact of ^68^Ga-PSMA PET/CT on radiation treatment planning of prostate cancer patients. Nuklearmedizin.

[55] Vorbach S.M., Rittmayer H., Seppi T., Nilica B., Kafka M., Ganswindt U. (2025). PSMA-PET/CT-based salvage elective nodal radiotherapy for lymph node recurrence following radical prostatectomy. World J. Urol..

[56] Fendler W.P., Calais J., Eiber M., Flavell R.R., Mishoe A., Feng F.Y., Nguyen H.G., Reiter R.E., Rettig M.B., Okamoto S. (2019). Assessment of ^68^Ga-PSMA-11 PET Accuracy in Localizing Recurrent Prostate Cancer. JAMA Oncol..

[57] Petit C., Delouya G., Taussky D., Barkati M., Lambert C., Beauchemin M.-C., Clavel S., Mok G., Paré A.-S.G., Nguyen T.-V. (2023). PSMA-PET/CT-Guided Intensification of Radiation Therapy for Prostate Cancer (PSMAgRT): Findings of Detection Rate, Effect on Cancer Management, and Early Toxicity From a Phase 2 Randomized Controlled Trial. Int. J. Radiat. Oncol. Biol. Phys..

[58] Sweeney C.J., Chen Y.-H., Carducci M., Liu G., Jarrard D.F., Eisenberger M., Wong Y.-N., Hahn N., Kohli M., Cooney M.M. (2015). Chemohormonal Therapy in Metastatic Hormone-Sensitive Prostate Cancer. N. Engl. J. Med..

[59] James N.D., De Bono J.S., Spears M.R., Clarke N.W., Mason M.D., Dearnaley D.P., Ritchie A.W.S., Amos C.L., Gilson C., Jones R.J. (2017). Abiraterone for Prostate Cancer Not Previously Treated with Hormone Therapy. N. Engl. J. Med..

[60] Lowrance W., Dreicer R., Jarrard D.F., Scarpato K.R., Kim S.K., Kirkby E., Buckley D.I., Griffin J.C., Cookson M.S. (2023). Updates to Advanced Prostate Cancer: AUA/SUO Guideline (2023). J. Urol..

[61] Calais J., Zhu S., Hirmas N., Eiber M., Hadaschik B., Stuschke M., Herrmann K., Czernin J., Kishan A.U., Nickols N.G. (2021). Phase 3 multicenter randomized trial of PSMA PET/CT prior to definitive radiation therapy for unfavorable intermediate-risk or high-risk prostate cancer [PSMA dRT]: Study protocol. BMC Cancer.

[62] Shetty D., Patel D., Le K., Bui C., Mansberg R. (2018). Pitfalls in Gallium-68 PSMA PET/CT Interpretation—A Pictorial Review. Tomography.

[63] Burger I., Sayyid R.K. APCCC Diagnostics 2025: False Positive PSMA PET: What to Know? 2026. https://www.urotoday.com/conference-highlights/apccc-diagnostics-2025/158672-apccc-diagnostics-2025-false-positive-psma-pet-what-to-know.html.

[64] Rizzo A., Morbelli S., Albano D., Fornarini G., Cioffi M., Laudicella R., Dondi F., Grimaldi S., Bertagna F., Racca M. (2024). The Homunculus of unspecific bone uptakes associated with PSMA-targeted tracers: A systematic review-based definition. Eur. J. Nucl. Med. Mol. Imaging.

[65] Grünig H., Maurer A., Thali Y., Kovacs Z., Strobel K., Burger I.A., Müller J. (2021). Focal unspecific bone uptake on [^18^F]-PSMA-1007 PET: A multicenter retrospective evaluation of the distribution, frequency, and quantitative parameters of a potential pitfall in prostate cancer imaging. Eur. J. Nucl. Med. Mol. Imaging.

[66] Barbosa F.d.G., Queiroz M.A., Nunes R.F., Costa L.B., Zaniboni E.C., Marin J.F.G., Cerri G.G., Buchpiguel C.A. (2020). Nonprostatic diseases on PSMA PET imaging: A spectrum of benign and malignant findings. Cancer Imaging.

[67] Paschalis A., Sheehan B., Riisnaes R., Rodrigues D.N., Gurel B., Bertan C., Ferreira A., Lambros M.B., Seed G., Yuan W. (2019). Prostate-specific Membrane Antigen Heterogeneity and DNA Repair Defects in Prostate Cancer. Eur. Urol..

[68] Mulati Y., Shen Q., Tian Y., Chen Y., Yao K., Yu W., Cui Y., Shi X., He Z., Zhang Q. (2025). Characterizing PSMA heterogeneity in prostate cancer and identifying clinically actionable tumor associated antigens in PSMA negative cases. Sci. Rep..

[69] Mantica G., Chierigo F., Ambrosini F., D’amico F., Celesti G., Ferrari A., Gallo F., Schenone M., Benelli A., Introini C. (2025). Interpretation of PSMA-PET Among Urologists: A Prospective Multicentric Evaluation. Cancers.

[70] Eiber M., Herrmann K., Calais J., Hadaschik B., Giesel F.L., Hartenbach M., Hope T.A., Reiter R., Maurer T., Weber W.A. (2018). Prostate Cancer Molecular Imaging Standardized Evaluation (PROMISE): Proposed miTNM Classification for the Interpretation of PSMA-Ligand PET/CT. J. Nucl. Med..

[71] Elsamny A., Wardeh A., Panyukova A., Kandel K., Lubin D., Nicola R. (2025). PSMA-RADS 2.0: A revised framework for PSMA-targeted imaging interpretation and clinical decision-making. Abdom. Radiol..

[72] Jochumsen M.R., Bouchelouche K. (2024). PSMA PET/CT for Primary Staging of Prostate Cancer—An Updated Overview. Semin. Nucl. Med..

[73] Wang Y., Jing R., Wang H., Zhao Q. (2024). ^68^Ga-PSMA-11 PET and mpMRI in the diagnosis of initial lymph node staging of prostate cancer: A head-to-head comparative meta-analysis. Front. Med..

[74] Privé B.M., Govers T.M., Israël B., Janssen M.J.R., Timmermans B.J.R., Peters S.M.B., de Groot M., Zámecnik P., Wijn S.R.W., Hoepping A. (2025). A cost-effectiveness study of PSMA-PET/CT for the detection of clinically significant prostate cancer. Eur. J. Nucl. Med. Mol. Imaging.

[75] Schwenck J., Rempp H., Reischl G., Kruck S., Stenzl A., Nikolaou K., Pfannenberg C., la Fougère C. (2017). Comparison of ^68^Ga-labelled PSMA-11 and 11C-choline in the detection of prostate cancer metastases by PET/CT. Eur. J. Nucl. Med. Mol. Imaging.

[76] Milojevic I.G., Milojevic B., Skrijelj D., Bumbasirevic U., Janicic A., Kajmakovic B., Sobic-Saranovic D., Artiko V., Beatovic S. (2025). The Role of Nuclear Medicine in Prostate Cancer. Diagnostics.

[77] Malaspina S., Anttinen M., Taimen P., Jambor I., Sandell M., Rinta-Kiikka I., Kajander S., Schildt J., Saukko E., Noponen T. (2021). Prospective comparison of ^18^F-PSMA-1007 PET/CT, whole-body MRI and CT in primary nodal staging of unfavourable intermediate- and high-risk prostate cancer. Eur. J. Nucl. Med. Mol. Imaging.

[78] Liu F.-Y., Sheng T.-W., Tseng J.-R., Yu K.-J., Tsui K.-H., Pang S.-T., Wang L.-J., Lin G. (2022). Prostate-specific membrane antigen (PSMA) fusion imaging in prostate cancer: PET–CT *vs* PET–MRI. Br. J. Radiol..

[79] Liu L.-L., Zhu L.-L., Lu Z.-G., Sun J.-D., Zhao J., Wang H.-F., Xiang Z.-L. (2023). Variability of radiotherapy volume delineation: PSMA PET/MRI and MRI based clinical target volume and lymph node target volume for high-risk prostate cancer. Cancer Imaging.

[80] Urso L., Badrane I., Manco L., Castello A., Lancia F., Collavino J., Crestani A., Castellani M., Cittanti C., Bartolomei M. (2025). The Role of Radiomics and Artificial Intelligence Applied to Staging PSMA PET in Assessing Prostate Cancer Aggressiveness. J. Clin. Med..

[81] Santucci D., Ragone R., Vergantino E., Vaccarino F., Esperto F., Prata F., Scarpa R.M., Papalia R., Zobel B.B., Grasso F.R. (2024). Comparison between Three Radiomics Models and Clinical Nomograms for Prediction of Lymph Node Involvement in PCa Patients Combining Clinical and Radiomic Features. Cancers.

[82] Yadav D., Hwang H., Qiao W., Upadhyay R., Chapin B.F., Tang C., Aparicio A., Lopez-Olivo M.A., Kang S.K., Macapinlac H.A. (2022). ^18^F-Fluciclovine versus PSMA PET Imaging in Primary Tumor Detection during Initial Staging of High-Risk Prostate Cancer: A Systematic Review and Meta-Analysis. Radiol. Imaging Cancer.

[83] Baratto L., Song H., Duan H., Hatami N., Bagshaw H.P., Buyyounouski M., Hancock S., Shah S.A., Srinivas S., Swift P. (2021). PSMA- and GRPR-Targeted PET: Results from 50 Patients with Biochemically Recurrent Prostate Cancer. J. Nucl. Med..

[84] Fernández R., Soza-Ried C., Iagaru A., Stephens A., Müller A., Schieferstein H., Sandoval C., Amaral H., Kramer V. (2023). Imaging GRPr Expression in Metastatic Castration-Resistant Prostate Cancer with [^68^Ga]Ga-RM2—A Head-to-Head Pilot Comparison with [^68^Ga]Ga-PSMA-11. Cancers.

[85] Seifert R., Emmett L., Rowe S.P., Herrmann K., Hadaschik B., Calais J., Giesel F.L., Reiter R., Maurer T., Heck M. (2023). Second Version of the Prostate Cancer Molecular Imaging Standardized Evaluation Framework Including Response Evaluation for Clinical Trials (PROMISE V2). Eur. Urol..

[86] Quarta L., Stabile A., Chiti A., Montorsi F., Briganti A., Gandaglia G. (2025). Radioguided Surgery for Prostate Cancer. Eur. Urol. Focus.

[87] Wen F., Schäfer L., Zheng X., Huang H., Noordzij W., Saar M., Mottaghy F.M., Lütje S. (2026). Diagnostic accuracy of PSMA-targeted radioguided surgery in prostate cancer at multiple anatomical levels: A systematic review and meta-analysis. Eur. J. Nucl. Med. Mol. Imaging.

[88] Schottelius M., Viertl D., Buckle T., Koch H., Martin S., Litvinenko A., Patt M., van Willigen D.M., van Leeuwen F.W.B., Wester H.-J. (2026). [^99m^Tc]Tc-PSMA-HSG for PSMA-targeted Hybrid Surgical Guidance: A new addition to the PSMA-I&S/I&T family. Eur. J. Nucl. Med. Mol. Imaging.

[89] Morris M.J., Castellano D., Herrmann K., de Bono J.S., Shore N.D., Chi K.N., Crosby M., Piulats J.M., Fléchon A., Wei X.X. (2024). ^177^Lu-PSMA-617 versus a change of androgen receptor pathway inhibitor therapy for taxane-naive patients with progressive metastatic castration-resistant prostate cancer (PSMAfore): A phase 3, randomised, controlled trial. Lancet.

[90] Hofman M.S., Emmett L., Sandhu S., Iravani A., Buteau J.P., Joshua A.M., Goh J.C., Pattison D.A., Tan T.H., Kirkwood I.D. (2024). Overall survival with [^177^Lu]Lu-PSMA-617 versus cabazitaxel in metastatic castration-resistant prostate cancer (TheraP): Secondary outcomes of a randomised, open-label, phase 2 trial. Lancet Oncol..

[91] Fizazi K., Chi K.N., Shore N.D., Herrmann K., de Bono J.S., Castellano D., Piulats J.M., Fléchon A., Wei X.X., Mahammedi H. (2025). Final overall survival and safety analyses of the phase III PSMAfore trial of [^177^Lu]Lu-PSMA-617 versus change of androgen receptor pathway inhibitor in taxane-naive patients with metastatic castration-resistant prostate cancer. Ann. Oncol..

